# Management of severe hyperinflammation in the COVID-19 era: the role of the rheumatologist

**DOI:** 10.1093/rheumatology/keaa652

**Published:** 2020-11-16

**Authors:** Charalampia Papadopoulou, Muthana Al Obaidi, Elena Moraitis, Sandrine Compeyrot-Lacassagne, Despina Eleftheriou, Paul Brogan

**Affiliations:** 1 Department of Paediatric Rheumatology, London, UK; 2 Infection, Inflammation and Rheumatology Section, London, UK; 3 NIHR Biomedical Research Centre, UCL Great Ormond Street Institute of Child Health, Great Ormond Street Hospital NHS Foundation Trust, London, UK; 4 Centre for Adolescent Rheumatology Versus Arthritis, London, UK

**Keywords:** hyperinflammation, paediatric inflammatory multisystem syndrome, SARS-CoV-2, clinical framework, multidisciplinary team

## Abstract

**Objectives:**

The objectives of this study were (i) to describe the clinical presentation, treatment and outcome of paediatric inflammatory multisystem syndrome temporally related to Sars-CoV-2 (PIMS-TS) in children; (ii) to propose a framework to guide multidisciplinary team (MDT) management; and (iii) to highlight the role of the paediatric rheumatologist in this context.

**Methods:**

This study involved a retrospective case notes review of patients referred to a single specialist paediatric centre with suspected PIMS-TS, with a focus on clinical presentation, laboratory parameters, treatment, and outcome in the context of an MDT framework.

**Results:**

Nineteen children of median age 9.1 years fulfilled the definition of PIMS-TS and were managed within an MDT framework: 5/19 were female; 14/19 were of Black, Asian or minority ethnicity; 9/19 also fulfilled diagnostic criteria for complete or incomplete Kawasaki disease (KD). Severe systemic inflammation, shock, and abdominal pain were ubiquitous. Treatment was stratified within an MDT framework and included CSs in all; i.v. immunoglobulin in all; anakinra in 4/19; infliximab in 1/19; and antiviral (aciclovir) in 4/19.

**Conclusions:**

We observed significant diagnostic equipoise using a current definition of PIMS-TS, overlapping with KD. Outside of clinical trials, an MDT approach is vital. The role of the paediatric rheumatologist is to consider differential diagnoses of hyperinflammation in the young, to advise on empiric immunomodulatory therapy, to set realistic therapeutic targets, to gauge therapeutic success, to oversee timely step-down of immunomodulation, and to contribute to the longer-term MDT follow-up of any late inflammatory sequelae.

**Rheumatology key messages**

- Current definitions of PIMS-TS do not reliably differentiate this entity from Kawasaki disease.
- Managing hyperinflammation in the COVID-19 era, outside of clinical trials, requires an MDT approach.
- The role of the paediatric rheumatologist is important in diagnosis, immunomodulatory therapy and longer-term follow-up.

## Introduction

Reports of a novel hyperinflammatory syndrome in children that could be related to severe acute respiratory syndrome coronavirus 2 (SARS-CoV-2) have emerged, possibly representing a rare, delayed immune-mediated response rather than direct viral sepsis in a small minority of children [1, 2]. On 1^st^ May 2020, the term PIMS-TS was proposed: paediatric inflammatory multisystem syndrome temporally related to SARS-CoV-2, defined as:

1. a child presenting with persistent fever, inflammation, and evidence of single or multi-organ dysfunction with additional features. This may include children fulfilling diagnostic criteria for Kawasaki disease (KD) [3, 4];
2. exclusion of any other microbial cause (waiting for results should not delay seeking expert advice);
3. SARS-CoV-2 PCR testing may be positive or negative [5].

This pragmatic case definition, formulated in the absence of any definitive diagnostic laboratory test, attempted to exclude other infectious causes of hyperinflammation, but never purported to differentiate patients with PIMS-TS from non-infectious hyperinflammatory states such as KD [3]. As such, considerable diagnostic confusion between PIMS-TS and KD has arisen, with early reports that PIMS-TS may represent a ‘KD-like’ illness [2]. This has confused clinicians, and worried patients (https://www.societi.org.uk/kawasaki-disease-covid-19/). 

KD is an acute self-limiting inflammatory medium-vessel vasculitis particularly affecting the coronary arteries. The aetiology of KD remains unknown [6–10]. It is currently proposed that any one of many infectious (or other wind-borne) triggers may result in an autoinflammatory response in genetically primed individuals [11, 12]. It is therefore a plausible hypothesis that SARS-CoV-2 may trigger KD in susceptible individuals, although this is as yet highly speculative and unproven.

In April 2020, as cases of PIMS-TS began to emerge in London, a multidisciplinary team (MDT) was established at Great Ormond Street Hospital NHS Foundation Trust to manage critically ill paediatric patients presenting with hyperinflammation. The group comprised key individuals from teams well versed in the use of immunomodulation to treat hyperinflammation in children: paediatric rheumatology; infectious disease; immunology; haematology; cardiology and intensive care. In view of the diagnostic and therapeutic uncertainties around PIMS-TS, the remit of this group was to:

1. formulate pragmatic clinical guidelines that gave consideration to the differential diagnosis of paediatric hyperinflammation; and to use these clinical guidelines to inform a practical pathway for therapeutic stratification;
2. propose and implement a practical therapeutic target to facilitate a treat-to-target approach: zero fever and zero CRP (i.e. <10 mg/l), a therapeutic target successfully used for KD [3];
3. advise on immunomodulation to achieve this therapeutic target in the context of recently proposed (non-evidence-based) guidance for the treatment of Covid-19 in the young [13].

The aims of this report were to summarize the clinical presentation, treatment, and outcome of cases discussed by this paediatric hyperinflammation MDT; to propose a clinical framework to help inform such MDT discussions; and to highlight the key roles of the paediatric rheumatologist in this context.

## Methods

### Patients

We retrospectively collated clinical data on consecutive cases discussed by our MDT between 4^th^ April 2020 (the date of the inaugural PIMS-TS MDT meeting) and 18^th^ May 2020. Medical notes were reviewed retrospectively with ethical approval. Since this was a retrospective case notes review of de-identified data, written informed consent was not required, as per national ethical requirements. We included patients (aged ≤18 years) with hyperinflammation and PIMS-TS as the possible cause, according to the Royal College of Paediatrics and Child Health (RCPCH) definition [5]. All patients were admitted to the paediatric intensive care unit (PICU), and all were tested for SARS-CoV-2 on admission by quantitative RT-PCR and/or serology; all underwent chest radiography.

### Data handling and statistics

Demographics, clinical features, virology status, and results of pertinent radiological and other laboratory parameters were summarized onto a de-identified Microsoft Excel spreadsheet (Version 16.0). Continuous variables were expressed as median and interquartile range (IQR), unless otherwise specified.

## Results

Demographics, clinical findings, treatment, and outcome for this cohort are summarized in **Table 1**. At the MDT meeting, 19 consecutive children were discussed who were of median age 9.1 years (IQR 7–13.8 years). Of the 19, 5 were females and 14 were of Black, Asian or minority ethnicity. Further imaging findings are provided in **Supplementary Table 1**, available at *Rheumatology* online. Of the 19, 3 had a notable past medical history: one had type 1 diabetes mellitus, one had sickle cell anaemia; and one had myelomeningocele and a ventriculoperitoneal shunt; no other comorbidities were reported in the remaining 16 patients. Seven children (36.8%) had positive SARS-CoV-2 RT-PCR on admission; 12 (63.2%) had positive SARS-CoV-2 antibodies; 3 had positivity both for RT-PCR and serology, while 17 had positive RT-PCR and/or serology. History of contact with known or suspected COVID-19 cases was established in 4 out of 9 cases. One/19 case was found to be positive for parainfluenza 2, also with positive SARS-CoV-2 antibodies; and 1/19 for adenovirus with negative SARS-CoV-2 RT-PCR and antibodies; and in 2/19 no evidence for viral or bacterial exposure was detected. For those with negative RT-PCR and serology, there was a high index of suspicion and similar phenotype.

**Table 1:** Demographic, clinical, and treatment characteristics of 19 patients discussed at the paediatric hyperinflammation MDT meeting

Pt	Age	Sex	SARS- CoV-2 PCR	SARS- CoV-2 ab	Fever duration days	GI	Rash	**Conju** **ctivitis**	Cracked lips	**IV Ig** **g/kg**	**i.v. MP/** **PO PD**	Anakinra	Inf	Hb	WCC	Neu	Lym	PLT	CRP	Fer	LDH	Fib	D-dimers	Trop	BNP	Dx
**1**	17.2	M	Pos		7	Y	Y	N	N	1	10 mg/kg*3	i.v. 2 mg/kg, then 4 mg/kg infusion		114	23.8	22.4	0.41	259	388	2026	715	6.7	2644	609	>35 000	PIMS-TS/ Incomplete KD
**2**	10.5	M	Neg	Pos	7	Y	N	Y	N	2	30 mg/kg*3			110	15.3	12.5	1.99	84	62	1595	1090	4.4	2366	520	7103	PIMS-TS/ Incomplete KD
**3**	8.7	M	Pos		9	Y	Y	Y	N	1	1.6 mg/kg	i.v. 2 mg/kg, then 4 mg/kg infusion		96	48.1	46.7	0.9	278	416	1005	617	6.3	2863	4135	>35 000	PIMS-TS/ Incomplete KD
**4**	10.1	F	Pos		10	Y	N	Y	N	2	10 mg/kg*3			108	23.3	21.7	0.95	194	270	2029	856	7	1992	70	2858	PIMS-TS/ Incomplete KD
**5**	8.9	M	Neg	Neg	3	Y	Y	Y	N	2	30 mg/kg*3			116	22.9	19.9	1.96	178	60	285	448	3.7	2227	124	12 760	PIMS-TS/ Incomplete KD
**6**	11.8	F	Pos	Pos	6	Y	N	Y	N	1	2 mg/kg			114	16.3	13.8	1.46	78	321	1529	600	4.7	1745	829	>35 000	PIMS-TS/ Incomplete KD
**7**	3.7	M	Neg		5	Y	Y	Y	Y	2	10 mg/kg*3			111	8.21	6.08	1.62	254	203	198	675	5.8	543	21	1515	PIMS-TS/KD
**8**	6.6	M	Pos	Pos	5	Y	N	Y	Y	1	30 mg/kg*3			87	31	27.8	1.83	44	223	4851	1130	4.4	2449	205	>35 000	PIMS-TS/ Incomplete KD
**9**	7.1	F	Neg	Pos	7	Y	Y	Y	N	2	10 mg/kg*3		5mg/kg	82	13.2	11.7	1.15	246	295	819	998	7.8	811	116	8122	PIMS-TS/ Incomplete KD
**10**	9	F	Neg	Pos	6	Y	Y	N	N	2	10 mg/kg*2, 30 mg/kg*3			97	19.5	17.3	0.86	274	361	1438	656	6.6	2282	274	31 137	PIMS-TS
**11**	9.1	F	Neg	Pos	2	Y	N	Y	Y	2	10 mg/kg*3			96	17	15.2	1.08	151	283	1118	845	6.9	2360	220	9639	PIMS-TS
**12**	14.4	M	Pos		5	Y	N	N	N	2	10 mg/kg*3	i.v. 2 mg/kg, then 4 mg/kg infusion		104	15.2	13.9	0.74	161	283	1675	764	6.7	2231	345	20 549	PIMS-TS
**13**	13.9	M	Neg	Pos	5	Y	Y	Y	Y	2	30 mg/kg*3	i.v. 2 mg/kg, then 2 mg/kg infusion		127	4.53	3.36	0.96	75	266	1835	821	5.9	2497	2996	4658	PIMS-TS
**14**	5.2	M	Neg	Pos	5	Y	N	N	N	2	10 mg/kg*3			99	17.3	9.66	5.67	597	54	426	2031	5.5	4459	25	2476	PIMS-TS/ sickle cell disease
**15**	14	M	Neg	Pos	5	Y	Y	Y	Y	2	10 mg/kg*3			124	8.42	5.5	1.39	178	180	250	578	4.9	543	167	3550	PIMS-TS
**16**	8.8	M	Pos	Pos	6	Y	Y	N	N	2	10 mg/kg*3			120	13.8	12.3	0.83	160	311	852	590	6.6	30001	570	24 825	PIMS-TS
**17**	14.7	M	Neg	Neg	3	Y	Y	Y	N	2	10 mg/kg*3			111	18.3	14.7	1.32	144	370	302	802	6.7	5603	975	4456	PIMS-TS
**18**	1	M	Neg	Neg	2	Y	Y	Y	N	2	2 mg/kg			107	11.5	8.7	1.56	98	161	551	1324	3.6	9604	27	5878	PIMS-TS
**19**	13.5	F	Neg	Pos	5	Y	N	N	N	2	10 mg/kg*3			92	12.8	10	1.26	194	301	190	1100	3.8	3154	12	521	PIMS-TS

BNP: brain natriuretic peptide; Dx: diagnosis; F: female; Fer: ferritin; Fib: fibrinogen; GI: gastrointestinal symptoms; Hb: haemoglobin; IVIg: Intravenous Immunoglobulin; Inf: infliximab; IV MP/PO PD: i.v. methylprednisolone/per os prednisolone; KD; Kawasaki disease; LDH: lactase dehydrogenase; Lym; lymphocytes; M; male; MDT: multidisciplinary team; Neg: negative; Neu: neutrophils; PIMS-TS: paediatric inflammatory multisystem syndrome temporally related to Sars-CoV-2; PLT: platelets; Pos: positive; Pt: patient; SARS-CoV-2 ab: severe acute respiratory syndrome coronavirus 2 antibodies; SARS-CoV-2 PCR: severe acute respiratory syndrome coronavirus 2 PCR; Trop: troponin; WCC: white cell count; Y/N: yes/no.

### Clinical features and diagnosis

All 19 patients fulfilled the RCPCH definition of PIMS-TS, but there was diagnostic equipoise for nine patients who also fulfilled diagnostic criteria for complete/incomplete KD (see below) [4]. All patients presented with high-grade temperature of median duration of 5 days (IQR 5–7 days); all patients had significant abdominal pain. Other clinical symptoms included diarrhoea in 14, rash in 10, red eyes in 13 (conjunctivitis being suspected clinically in all of them), cervical lymphadenopathy in 2, and cracked lips in 4. Of the 19, 17 (89%) progressed rapidly to develop warm, vasoplegic shock, refractory to volume resuscitation and eventually requiring noradrenaline and milrinone for haemodynamic support. Ten (52%) required mechanical ventilation for a median of 3 days (IQR 1–4.0). Abdominal US was performed at the time of admission on all 19 patients: in 5, this revealed evidence of colitis. A total of 14 (73%) had evidence of cardiac involvement (**Supplementary Table 1**, available at *Rheumatology* online), with increased troponin I, median 274 ng/l [IQR 116–609; reference range (RR) <34 ng/l] and brain natriuretic peptide, median 7103 pg/ml (IQR 3550–20549; RR 29–206 pg/ml). One patient with sickle cell disease had coronary artery aneurysms (CAAs), with a *z* score for the right coronary artery of 4.2, and a *z* score for the left coronary artery of 6.4 [14, 15]. One patient required extracorporeal membrane oxygenation for refractory shock secondary to poor Left ventricular (LV) function and myocarditis. All patients had evidence of hyperinflammation, with grossly elevated CRP, median 283 mg/l (IQR 180–321 mg/l; reference range [RR] <20 mg/l); median ferritin 1005 μg/l (IQR 388–1675; RR 4.2–62.0 μg/l); median fibrinogen 5.9 g/l (IQR 4.7–6.7; RR 1.7–4.0 g/l); and median D-dimers 2366 μg/l (IQR 1992–3154; RR 0–312 μg/l). When patients with PIMS-TS without KD features were compared with the 9 patients who also fulfilled diagnostic criteria for complete/incomplete KD, no significant differences were identified in regard to demographic, clinical or laboratory features.

### Pragmatic stratified treatment framework

**Fig. 1:**
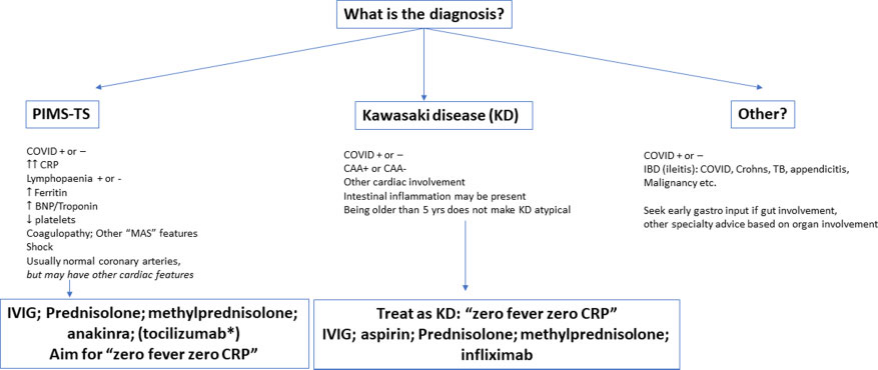
Pragmatic stratified therapeutic approach for PIMS-TS in an expert MDT setting PIMS-TS: paediatric inflammatory multi-system syndrome temporally related to SARS-CoV-2; MDT: multidisciplinary team; COVID: coronavirus disease; BNP: brain natriuretic peptide; MAS: macrophage activation syndrome; CAA: coronary artery aneurysm; TB: tuberculosis; gastro: gastroenterology. *Tocilizumab in parentheses, since not used in our series of patients, but is being explored in clinical trials of Covid-19, so may be a therapeutic option for PIMS-TS.

All 19 patients were discussed at the Covid-19 MDT meeting upon admission, and consensus was reached regarding the final pragmatic clinical diagnosis for informing a stratified therapeutic approach, summarized in Fig. 1. Based on that MDT opinion, the final pragmatic clinical diagnoses were 10 patients with PIMS-TS without KD; and a diagnostic equipoise overlap group of 9 patients fulfilling both the proposed definition of PIMS-TS [5] and also diagnostic criteria of complete or incomplete KD [4]. Non-mutually exclusive immunomodulatory therapeutics deployed based in this diagnostic framework were therefore: (i) IVIg 2 g/kg (*n* = 16), or 1 g/kg (*n* = 3); (ii) off-label use of anakinra (s.c. or i.v.), based on a case-by-case discussion of inflammation severity; (iii) infliximab (6 mg/kg i.v.) for those with suspected IVIg/CS-resistant KD (*n* = 1). In addition to these immunomodulators, all patients received concomitant CSs: 15 patients received pulses with i.v. methylprednisolone [(MP); 10–30 mg/kg; maximum dose 1 g] for 3 consecutive days; 4 were given oral prednisolone 2 mg/kg/day (or i.v. MP equivalent 1.6 mg/kg/day), followed (for all patients) by oral prednisolone 2 mg/kg/day until the CRP normalized to <10 mg/l. CSs were then weaned off over 3–4 weeks. Regarding the use of anakinra, 4 patients with suspected PIMS-TS (but without suspicion of KD) received this at a dose of 2 mg/kg i.v. bolus, followed by 4 mg/kg/day as a continuous i.v. infusion in 3 of those 4, and 2 mg/kg as a continuous i.v. infusion in the fourth patient. We also implemented the following step-down dosing regimen for those on i.v. anakinra once CRP had normalized: switch to 2–4 mg/kg anakinra s.c. (max 100 mg i.e. one vial) for 3 days; then 1 mg/kg for 3 days (max 50 mg i.e. half a vial); then stop (monitor CRP daily while weaning anakinra).

Of the 19, 9 patients also received anti-aggregation therapy; 12 of the 19 received prophylactic low-molecular-weight heparin. All were given empiric broad-spectrum antibiotics, and 4 received additional empiric anti-viral treatment with aciclovir.

### Outcome

All children survived and were discharged from PICU after a median of 3 days (IQR 1–6 days). The median time to resolution of fever and normalization of CRP was 10 days (IQR 7.8–11 days). At the latest follow-up post discharge (median 8 days, IQR 5–17 days), cardiac function had completely normalized in all patients; one patient had persistent CAA (coronary artery *Z* score >2.5), although this patient had sickle cell disease, a condition known to be associated with coronary artery dilatation [14]. At the time of writing, an MDT follow-up clinic is being established to monitor for any cardiac or inflammatory deterioration as CSs are weaned.

## Discussion

Our retrospective analyses confirm the occurrence of a hyperinflammatory syndrome temporally related to SARS-CoV-2. We demonstrated significant diagnostic equipoise using the current RCPCH case definition of PIMS-TS, particularly in relation to KD, since nine patients in our series also fulfilled diagnostic criteria for KD. This might be explained by an immunological stimulus such as superantigens, as has been proposed by some [16, 17], resulting in a toxic shock phenotype (which is known to overlap with some features of KD); or the impact of a virus with tropism for endothelium, resulting in acute systemic vasculitis reminiscent of some features of KD in young children [18, 19]; or some other reason. Future studies will hopefully elucidate this. The PIMS-TS syndrome in this series, however, had important differences from historical cases of KD, notably the older age of the patients; the greater severity and/or frequency of systemic inflammation and cardiac shock; the need for PICU and/or inotropic support in most patients; and the presence of abdominal pain as a prominent clinical feature in all cases. Toxic shock syndrome and macrophage activation syndrome (with or without KD) are also very important differential diagnoses in this context, and the clinician needs to be ever alert to these possibilities.

Given the diagnostic and therapeutic uncertainty around PIMS-TS, and the lack of any clinical trial open to children, out of necessity we devised a framework to facilitate the MDT management of these complex cases, and attempted to treat each case in a pragmatic and stratified way within that framework (Fig. 1). We used a variety of anti-inflammatory and immunomodulatory therapies **(****Table 1****;**Fig. 1**)** aimed at switching off the severe systemic inflammation, and adopted a therapeutic target borrowed from our experience of treating KD, which aims for rapid suppression of systemic inflammation by aiming for ‘zero fever and zero CRP’, i.e. resolution of fever and normalization of CRP as markers of therapeutic success [3]. Using this approach, irrespective of any diagnostic debate around KD *vs* PIMS-TS, survival was 100%; cardiac function fully recovered in all, with CAA in only one patient who also had sickle cell disease, a condition that is well established to also cause coronary changes. Furthermore, there were no infectious adverse events arising from using immunosuppression, which strongly suggests that the hyperinflammation of PIMS-TS stems from a dysregulated immune-mediated host response rather than fulminant viral sepsis *per se*. Of considerable concern, however, is that thrombotic complications were observed in two patients (Table 1). This teaches us that anti-thrombotic strategies, as well as immunomodulation, need to be built into the design of any future clinical trials designed for children with PIMS-TS.

We advocate that a specific paediatric trial for the sickest paediatric patients with PIMS-TS may need to be urgently designed, particularly in anticipation of a second wave of pandemic infection. This poses significant challenges which include: the now very clear and urgent need for a stricter case definition of PIMS-TS to be used as an inclusion criterion to avoid diagnostic confusion with KD; the likely need to adopt a rare disease trial design, such as a Bayesian approach [20], since patient numbers are significantly smaller than adult hyperinflammatory disease; and the important question regarding the best therapeutic approach for PIMS-TS to fully address the complex pathogenesis.

In conclusion, we confirm the occurrence of a hyperinflammatory state, probably induced by SARS-CoV-2 infection, in a small minority of children, and suggest that this entity is distinct from KD with important but different cardiac sequelae, probably representing myocardial stunning from a pro-inflammatory cytokine storm rather than direct viral myocarditis. We emphasize that current definitions of PIMS-TS do not reliably differentiate this entity from KD, and that this will have important therapeutic consequences for clinical decision making and future trial design. We propose a pragmatic, stratified therapeutic approach to aid the management of suspected PIMS-TS **(**Fig. 1**)**. Thus, managing hyperinflammation of the young in the COVID-19 era demands considerable clinical acumen, and outside of clinical trials it requires an MDT approach. We suggest that the role of the paediatric rheumatologist is to consider important differential diagnoses of non-infectious hyperinflammation; to advise on immunomodulatory therapy and set realistic targets to gauge therapeutic success; to oversee timely step-down of immunomodulation when inflammation has resolved; and to contribute to the longer-term MDT follow-up of any late inflammatory sequelae.

## Supplementary Material

keaa652_Supplementary_Data
