# Supplementary material for: Management of severe hyperinflammation in the COVID-19 era: the role of the rheumatologist
Source: Rheumatology (Oxford). 2020 Nov 16;60(2):911–7. doi: 10.1093/rheumatology/keaa652 (PMC7717388; doi:10.1093/rheumatology/keaa652)
Supplement: keaa652_Supplementary_Data [file keaa652_supplementary_data.docx]

| **Supplementary Table 1. Radiological findings** | | | | | |
| --- | --- | --- | --- | --- | --- |
| **Pt** | **Chest X/Ray** | **CT/US abdomen** | **Echocardiogram** | **MRI/CT brain** | **Complications** |
| **1** | Bilateral interstitial pneumonia | Sigmoid colitis | Impaired biventricular function, prominent coronary arteries |  | Inferior vena cava thrombus |
| **2** | Mild perihilar opacification | Normal | Normal |  |  |
| **3** | Perihilar airspace changes. Small bilateral apical effusions. Mild left retrocardiac consolidation. | Inflammatory changes in the right iliac fossa (echogenic fat + mildly thickened small bowel) with free fluid containing fine debris + some septations | Mild- moderately impaired systolic LV function (in context of high inotropic support) with more pronounced hypokinesia in apical region (FS 25- 27%). LV mild + dilated  Good overall RV systolic function Both AV valves with some accessory tissue, thin leaflets and trivial TR/ MR. Tiny rim of pericardial fluid | Resolving lesions of the corpus callosum with residual changes of genu and splenium. with suspicion of pus present in the occipital horns and the size of the ventricles has slightly increased. Ventriculitis |  |
| **4** | Atelectasis in the left lower lobe |  | Impaired biventricular function |  |  |
| **5** | Mild perihilar and basal bronchial wall thickening |  | Small pericardial effusion localised to the LV and RA. Evidence of myo/pericarditis. Mild LV systolic dysfunction and evidence of diastolic dysfunction. Evidence of pulmonary hypertension |  |  |
| **6** | Left lung consolidation  Small right pleural effusion with diffuse perihilar/midzone and lower lobe opacification | Free fluid and periportal oedema. The caecum and ascending colon appeared inflamed | Severe LV, globally impaired function, moderately dilated. Moderate MR. Trivial AR |  |  |
| **7** | Normal |  | No coronary dilatation |  |  |
| **8** | Left lower consolidation. Some perihilar airspace shadowing bilaterally | Pelvic free fluid with some mesenteric inflammation | LV systolic function mildly impaired (FS 27%) Tiny rim of fluid around LV (not significant) Prominent appearance RCA (measurements of coronary arteries remains in normal range- BOSTON data z-score).  No localised coronary artery aneurysms |  |  |
| **9** | The lungs are well expanded and clear | Small amount of free fluid in the right iliac fossa | Hypertrabeculated LV- systolic function borderline, mild impairment (FS 29-31%) Mild MR and subjectively mildly dilated LA (z-score remains normal) Prominent/ ectatic LCA, no localised aneurysms/ clots  No pericardial effusion |  |  |
| **10** | Consolidation in the left lower lobe and a right-sided pleural effusion | Generalised mesenteric fat inflammation with a large volume of free fluid but no focal collection | Mild systolic impairment | Leptomeningeal inflammation | Weakness/numbness Right lower limb and new Left sided squint |
| **11** | Mild increased airspace opacification in the right lower zone, with small right basal effusion |  | Mild+ MR.  Mild TR. Mildly dilated LV with moderately impaired LV systolic function.  Mild to moderately impaired RV systolic function. |  |  |
| **12** | Normal | Inflammatory changes in ileo-caecal region with associated lymphadenopathy | Moderate TR | Diffuse cerebral and cerebellar volume loss | Focal left hand weakness |
| **13** | Bilateral pleural effusions with left lower lobe collapse/consolidation | Trace amount of fluid noted in the right iliac fossa | Structurally normal heart Good biventricular function No pericardial effusion No overt aneurysmal coronary or aortic dilatations |  |  |
| **14** | Clear pleural spaces.  Enlarged heart |  | LMCA enlarged 7 mm (z-score + 8.9) at the ostium and 4 mm after. RCA enlarged 5 mm (z-score + 6.2) |  | PRES |
| **15** | The heart and mediastinum are normal. The lungs are clear. |  | Structurally normal heart with good cardiac function The LMCA looks mildly dilated visually but z score is within normal |  |  |
| **16** | Peribronchial infiltration predominantly in the right perihilar distribution. Minor left basal atelectasis. Blunting of the right costophrenic angle | Large volume of free fluid in the abdomen and pelvis | Mildly impaired LV systolic function Mild MR normal coronaries |  |  |
| **17** | No focal lung infiltrate | Trace free fluid within the pelvis | Mildly impaired LV systolic function, normal coronaries, no pericardial effusion |  |  |
| **18** | Small apical pleural effusions are noted. The lungs are clear | Thick-walled oedematous gallbladder and small bowel with ascites | Mild MR Normal Cardiac Function | Bifrontal multiple small foci of diffusion restriction - Peripheral distribution favours embolic ischemic changes |  |
| **19** | The lungs are clear | Unremarkable ultrasound examination of the abdomen | Normal cardiac function Normal coronary arteries No pericardial effusion |  |  |
| Abbreviations. AR: Aortic regurgitation; AV: Atrioventricular valves; CT: Computed tomography; FS: Fractional shortening; LCA: Left coronary artery; LMCA: Left main coronary artery; LV: Left ventricle; MR: Mitral regurgitation; MRI: Magnetic resonance imaging; PRES: Posterior reversible encephalopathy syndrome; PT; Patient; RA: Right atrium; RCA: Right coronary artery; RV: Right ventricle; TR: Tricuspid regurgitation; US: Ultrasound | | | | | |
